# Military Personals

**Published:** 1918-01

**Authors:** 


### MILITARY PERSONALS

To Army Medical School, Washington, D. C., Lieut. Francis J. A. Bennett, Auburn.

To Camp Custer, Battle Creek, Mich., Capt. Alfred W. Armstrong, Canandaigua.

To Camp Dix, Wrightstown, N. J., Lieut. Harold F. Mickley, Seneca Falls.

To Chicago Northwestern University Dental School, Lieuts. George S. Skiff, Gainesville; Frederick W. Lester, Seneca Falls.

To Fort Oglethorpe, Lieut. Frederick C. Devendorf, Utica.

To Fort Sam Houston, Texas, Lieut. Francis T. Duffy, Troy.

To Ithaca, Cornell University, Capt. Samuel A. Munford, Ithaca.

To New Orleans, La., Charity Hospital, Lieut. Edward P. Flood, Albany.

To New York City, Roosevelt Hospital, Capt, Archer D. Babcock, Randolph.

To Camp Lee, Petersburg, Va., Lieut. Francis Argus, Buffalo.

To Camp Upton, Yaphank, L. I., Capts. Walter H. Vosburg, Dunkirk; Joseph R. Culkin, Rochester.

To New York City, U. S. Army General Hospital No. 1, Lieuts. Myer S. Bloom, Binghamton; Edwin C. Foster, Penn Yan.

To Camp Sherman, Chillicothe, Ohio, Lieuts. Byron C. Shults, Albany; Howard C. Murphy, Herkimer; Albert C. Burand, Ithaca.

To Fort Banks, Mass., Lieut. William J. Wansboro, Albany.

To Fort Niagara, Lieut. Edward F. Gillick, Niagara Falls.

To Fort Riley, Kan., Lieut. Roy J. Marshall, Rome.

To New York City, for special hospital work, Major Marin B. Tinker; Capt. George S. Britten, Syracuse; Maurice D. Barnette, Watertown.

To Plattsburg Barracks, for duty in connection with the examination of candidates for aviation, Capt. George F. Cott, Buffalo; Lieut. Edward F. Meister, Buffalo.

To Rochester, Minn., Lieuts. Frank A. Walder, Lockport; Paul M. Parker, Rochester; Philip Gordon, Rochester.

To his home and the inactive list on account of being physically disqualified for active service, Lieut. Samuel W. Houston, Walcott.

Capt. Charles E. Long of Buffalo has been transferred to the Mobilization Camp Aviation Section Signal Corps, Camp Sheridan, Montgomery, Ala.

